# Metabolic engineering of folate and its precursors in Mexican common bean (*Phaseolus vulgaris* L.)

**DOI:** 10.1111/pbi.12561

**Published:** 2016-04-25

**Authors:** Naty G. Ramírez Rivera, Carolina García‐Salinas, Francisco J.L. Aragão, Rocío Isabel Díaz de la Garza

**Affiliations:** ^1^ Tecnologico de Monterrey Escuela de Ingeniería y Ciencias Monterrey Nuevo León México; ^2^ Embrapa Recursos Genéticos e Biotecnologia Brasília Distrito Federal Brazil

**Keywords:** biofortification, folate, common bean, metabolic engineering, GTP cyclohydrolase I, micronutrients

## Abstract

Folate (vitamin B9) deficiency causes several health problems globally. However, folate biofortification of major staple crops is one alternative that can be used to improve vitamin intakes in populations at risk. We increased the folate levels in common bean by engineering the pteridine branch required for their biosynthesis. GTP cyclohydrolase I from Arabidopsis (*AtGchI*) was stably introduced into three common bean Pinto cultivars by particle bombardment. Seed‐specific overexpression of AtGCHI caused significant increases of up to 150‐fold in biosynthetic pteridines in the transformed lines. The pteridine boost enhanced folate levels in raw desiccated seeds by up to threefold (325 μg in a 100 g portion), which would represent 81% of the adult recommended daily allowance. Unexpectedly, the engineering also triggered a general increase in PABA levels, the other folate precursor. This was not observed in previous engineering studies and was probably caused by a feedforward mechanism that remains to be elucidated. Results from this work also show that common bean grains accumulate considerable amounts of oxidized pteridines that might represent products of folate degradation in desiccating seeds. Our study uncovers a probable different regulation of folate homoeostasis in these legume grains than that observed in other engineering works. Legumes are good sources of folates, and this work shows that they can be engineered to accumulate even greater amounts of folate that, when consumed, can improve folate status. Biofortification of common bean with folates and other micronutrients represents a promising strategy to improve the nutritional status of populations around the world.

## Introduction

**Figure 1:**
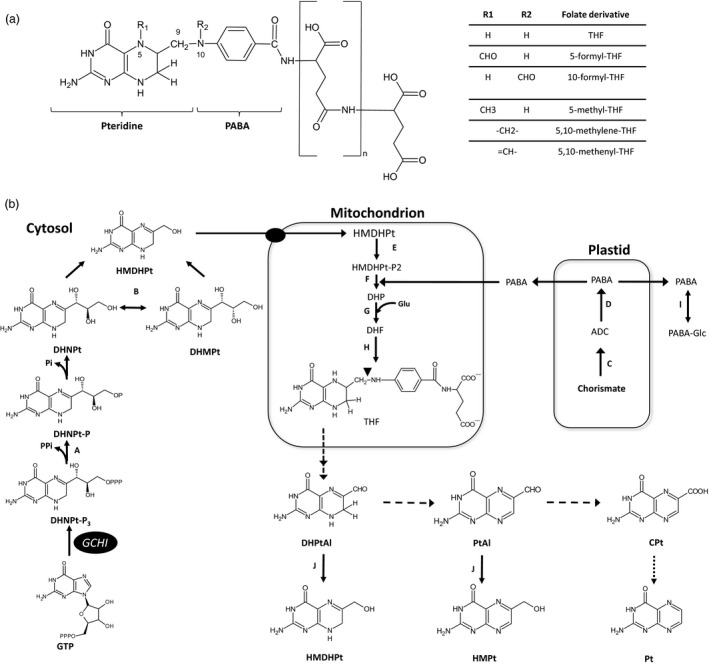
Folate derivatives and its biosynthesis in plants. Chemical structure of polyglutamylated folates, ‐n represents the number of glutamates attached to the first glutamate. R1 and/or R2 attached to N5 and/or N10 yield different species of folates (Panel a). Biosynthesis and oxidation of folates in plants (Panel b). GTP cyclohydrolase I (GCHI); A, dihydroneopterin (DHNPt) triphosphate diphosphatase; B, DHNPt aldolase; C, aminodeoxychorismate synthase (ADCS); D, aminodeoxychorismate lyase; E, 6‐hydroxymethyldihydropterin (HMDHPt) pyrophosphokinase; F, dihydropteroate (DHP) synthase; G, dihydrofolate (DHF) synthase; H, DHF reductase; I, UDP‐glucose–*p*‐aminobenzoate glucosyltransferase; J, NADPH‐dependent pterin aldehyde reductase. Dark circle, inferred transporter. Abbreviations: ADC, aminodeoxychorismate; CPt, 6‐carboxypterin; DHMPt, dihydromonapterin; DHPtAl, dihydropterin‐6‐aldehyde; GTP, guanosine‐5′‐triphosphate; HMPt, 6‐hydroxymethylpterin; ‐P, phosphate; PABA, p‐aminobenzoate; PABA‐Glc, p‐aminobenzoate β‐D‐glucopyranosyl ester; Pt, pterin; PtAl, pterin 6‐aldehyde; THF, tetrahydrofolate. Dashed arrows indicate photochemical oxidation steps; dotted arrows indicate possible oxidation (Rokos *et al*., 2002). Figure adapted from Hanson and Gregory (2011).

Common bean (*Phaseolus vulgaris* L.) is a grain legume that contains high amounts of energy and is a good source of minerals and vitamins. Bean grains are also rich in protein and represent the major source for some populations. Common bean is also the third most important source of calories in Latin America (Broughton *et al*., 2003). The annual worldwide production of this legume in 2013 was more than 23 million metric tonnes. Brazil is the largest bean producer in the Americas, followed by Mexico and the United States of America, while the major producers worldwide are found in Asia (Myanmar and India, respectively) (FAO, 2013). Humans require a minimum daily intake of essential micronutrients to fulfil the biochemical processes needed for life, and micronutrient deficiency is a major global problem; around 40%–50% of the world's population will at some point in their lives suffer from a disorder caused by a mineral and/or vitamin deficiency (Graham *et al*., 2001). Folates (vitamin B9) are essential components in the human diet; they are involved in several anabolic pathways that require one‐carbon (1C) transfer reactions such as nucleotide and amino acid biosynthesis, the methylation cycle and NADPH production (Bekaert *et al*., 2008; Fan *et al*., 2014). Humans cannot synthesize folates as plants and bacteria can; thus, we need to obtain them from the diet and plants are our main source. Folate is a generic term that encompasses tetrahydrofolate (THF) and its derivatives (Figure 1a). A THF molecule is composed of a pteridine ring that is synthesized in cytosol, p‐aminobenzoate (PABA) is produced in plastids, and both are condensed and glutamylated in mitochondria (Figure 1). In addition, natural folates have a γ‐linked polyglutamyl tail that is added by the folylpolyglutamate synthase (FPGS).

Folate malnutrition is a worldwide problem and is associated with the onset of megaloblastic anaemia (Rush, 2000), neural tube defects (NTD, Bailey *et al*., 2015), an increased risk of cardiovascular disease, certain cancers (McNulty and Scott, 2008) and neuropsychiatric disorders (Araújo *et al*., 2015). Dietary folate deficiency has been reduced in some countries by the addition of chemically synthesized folic acid to staple foods or by supplementation (Quinlivan and Gregory, 2007). However, these strategies rely on food processing and distribution chains that are not always available to all populations. A promising alternative is folate biofortification in plant foods. Biofortification enhances a food's nutritional quality through breeding or engineering; it has the potential to be sustainable and, if conducted in major staple crops, reach targeted populations (Mayer *et al*., 2008).

Different folate biofortification efforts have been conducted in various plant platforms over the past few years. The first proofs of concept were performed using *Arabidopsis* (Hossain *et al*., 2004) and tomato (Diaz de la Garza *et al*., 2004, 2007). Folate engineering has also been conducted in rice (Storozhenko *et al*., 2007), lettuce (Nunes *et al*., 2009), maize (Naqvi *et al*., 2009) and potato (Blancquaert *et al*., 2013). All previous works were accomplished by overexpressing one or two committed steps for folate biosynthesis: the synthesis of dihydroneopterin triphosphate from GTP and aminodeoxychorismate from chorismate. These reactions are mediated by GTP cyclohydrolase I (GCHI) and aminodeoxychorismate synthase (ADCS), respectively (Figure 1b). Recently, the expression of FPGS and folate‐binding protein along with GCHI and ADCS has further increased folates in rice endosperm (Blancquaert *et al*., 2015). Biofortification of plant foods with folates can be feasible, and common bean is both a good source and vehicle for nutrient delivery in Latin America and elsewhere. This work reports the first engineering of folates in a legume plant. Three common bean varieties, cv. Pinto Saltillo, cv. Pinto Café and cv. Pinto Durango (S, C and D), were transformed to overexpress *Arabidopsis thaliana* GTP cyclohydrolase I (AtGCHI), which induced an increase in pteridine, PABA and folate levels.

## Results

### Common bean stable transformation by particle bombardment

Common bean was transformed by particle bombardment, the only technique that has successfully produced stable transgenic lines in this species (Aragão *et al*., 2002). Two thousand four hundred embryonic axes from each cultivar were bombarded with the pAHAS‐AtGCHI vector (Supplementary Figure S1) containing the genes *AtGchI* and *ahas* (which confers resistance to imidazoline herbicides) under seed‐specific and constitutive promoters, respectively. After *in vitro* selection, plantlets were acclimated in soil and 18 were PCR positive (PCR^+^) for the genes *AtGchI* and *ahas*. Genomic DNA from alternate leaves and seeds of each plant was obtained and tested to assure that all tissues were transformed, as this technique could generate chimeric plants; both recombinant genes were amplified in all cases, ensuring transmission to the next generations (not shown). Transformation frequency (%) for each Pinto cultivar was measured as the number of fertile transgenic plants/number of embryonic axes bombarded; these frequencies were 4.15% (S), 2.5% (C) and 0.41% (D). The frequency of co‐transformation of *AtGchI* and *ahas* was 100% for all plants tested. Regarding visual phenotype, wild type, and T_0_ PCR^+^ plants had the same average pods per plant, 15 pods‐S, 17 pods‐C and 20 pods‐D. The number of seeds per pod was 3.11 ± 0.15 SE for each of the three cultivars.

The seeds from T_0_ plants were advanced to ensure genotype and phenotype maintenance. Five T_1_ seeds were germinated per independent line observing 100% germination. All T_1_ plants were PCR^+^, and T_2_ seeds from these plants were used to grow a larger number of plants to have enough seed material for full characterization (T_3_ generation). This progeny also had 100% germination, and all plants contained both transgenes. T_3_ seeds were advanced to T_4_ plants. Of the 20 lines advanced, five lines produced progeny that were 100% PCR^+^ while the other 15 segregated differently (20%–90%), thus confirming the stability of the transformation. Both transgenes also co‐segregated in all T_4_‐screened plants. Pteridines were analysed in T_1_ seeds for preliminary evidence of the engineering, and complete metabolite and expression analyses were performed on T_3_ seeds.

### PCR^+^ AtGCHI T_1_ bean seeds have significant increases in total pteridines

**Figure 2:**
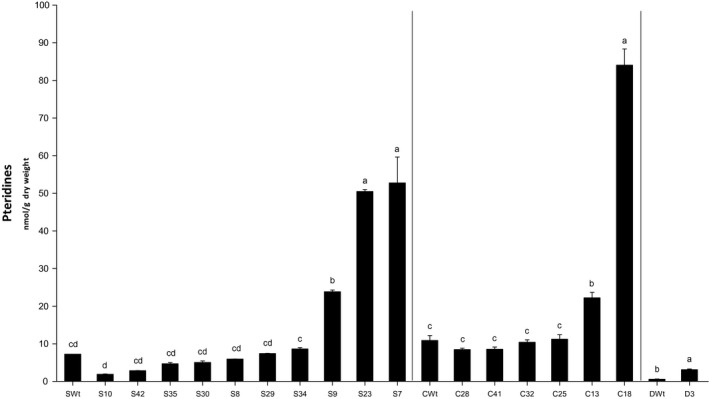
Total pteridine accumulation in AtGCHI‐expressing T_1_ bean seeds. Pteridine contents were quantified using seeds from each primary transgenic line and compared to Wt seeds. Values are means of three independent seed samples; error bars indicate standard error (SE). Letters indicate statistical difference determined using Student′s *t*‐test (*P* < 0.05). Comparisons were made per variety.

To different extents, total pteridines increased significantly in some of the AtGCHI seeds. Three Saltillo primary transformants had significantly higher levels of total pteridines; this was also found in two Café and in the Durango‐only transgenic lines (Figure 2). This shows that AtGCHI was correctly expressed and drove increases in the pteridine pool in the bean seeds. The highest pteridine accumulators, S7 and C18, contained 52.8 and 84 nmol/g dry weight (DW) of pteridines, which represented seven times more than the Wt lines. On the other hand, D3 seeds accumulated five times as many pteridines as DWt.

### Pteridine characterization in Pinto and T_3_ bean seeds

**Figure 3:**
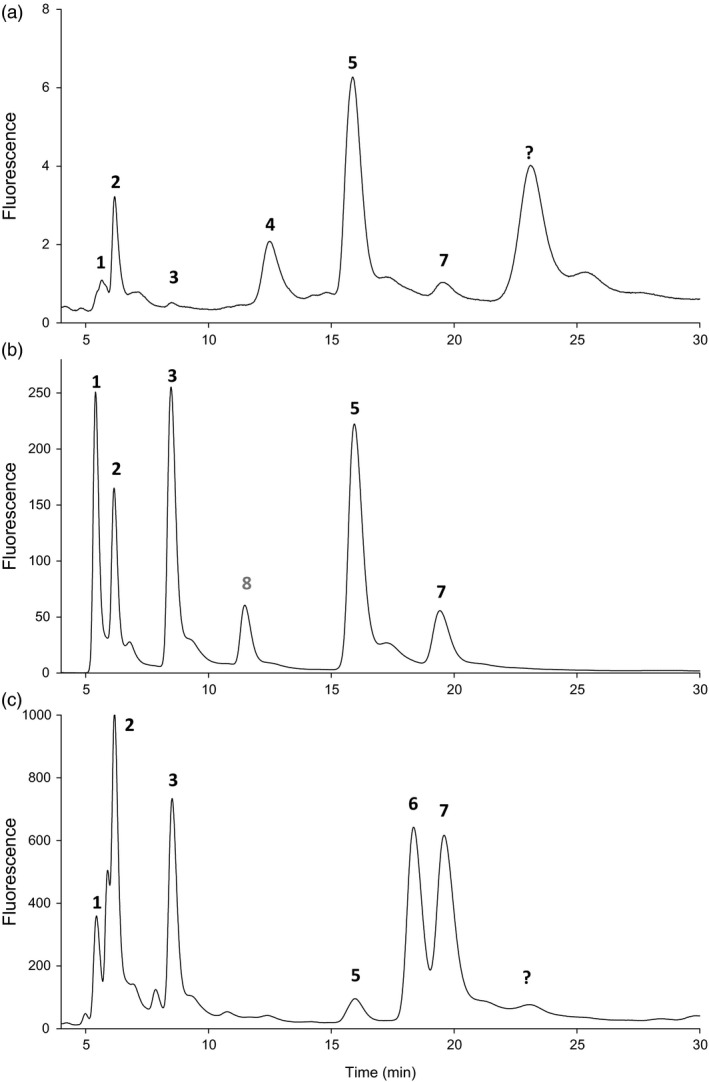
Pteridine profiles in Pinto bean seeds. Oxidized pteridines from Pinto Café wild‐type seeds (Panel a). Profile of pteridine standards added to a Pinto Café extract (Panel b). Pteridine profile of C18 engineered line (Panel c). CPt, 6‐carboxypterin (1); NPt, neopterin (2); MPt, monapterin (3); NPt‐G, neopterin glycoside (4); Pt, pterin (5); PtAl, pterin 6‐aldehyde (6); HMPt, 6‐hydroxymethylpterin (7); XPt, xanthopterin (not detected in seed extract) (8); unknown peak (?).

Pteridine profiles are shown in Figure 3; peak identification was based on retention time and comparison with true standards (Figure 3b). There was no previous knowledge about pteridine profiles in Pinto beans; thus, the Wt varieties used in this study were characterized first. A typical Wt pteridine profile is shown in Figure 3a. Pterin (Pt) and neopterin (Npt) were the major pteridines detected (53% and 20% of the pool); the former can be a product of folate degradation while the latter is a folate precursor (Figure 1b). For better interpretation, characterized pteridines were grouped into two classes: those involved in folate biosynthesis (Table 1) and those products of folate breakdown (Table 2). Concerning biosynthetic pteridines, Wt bean seeds accumulated very small amounts of 6‐hydroxymethylpterin (HMPt), the immediate precursor of folates. It represented only up to 2% of the pool. Npt and its stereoisomer, monapterin (Mpt), can also occur as glycosylated forms (Diaz de la Garza *et al*., 2004) and Wt seeds accumulated Npt‐glycoside (Npt‐G), especially Pinto Durango. Npt‐G peak was assigned (Figure 3) and confirmed by its disappearance when the sample was subject to acid hydrolysis following an increase in the Npt peak (not shown). However, Mpt‐glycoside could not be detected in any of the Wt extracts; in fact, Mpt accumulated in minute amounts in the bean seeds. In contrast to the biosynthetic pteridines, those products of folate breakdown (mostly Pt) represented up to 73% of the pool (Table 2). Additional peaks in the chromatogram that could be pteridines were detected; however, they were minor contributors to the pool (<3.0%). For example, an ‘unknown’ peak (Figure 3a) was predominant in all three Wt seeds, but represented a minor component in transgenic extracts (Figure 3c); by contrast, other minor peaks increased as did characterized pteridines in AtGCHI overexpressors. Thus, there are probably more pteridines present in bean seeds; however, here we are simply characterizing and considering the major components of the pool.

**Table 1:** Biosynthetic pteridines in Pinto seeds

Pteridine content (nmol/g dry weight)					
	NPt	MPt	NPt‐G	HMPt	Total biosynthesis
**SWt**	**0.8 ± 0.0**	**0.1 ± 0.0**	**0.3 ± 0.0**	**0.1 ± 0.0**	**1.2 ± 0.0**
S30	0.8 ± 0.0	0.1 ± 0.0	0.3 ± 0.0	0.1 ± 0.0	1.3 ± 0.0
S8	1.6 ± 0.0**	Traces	0.9 ± 0.0**	<0.05	2.5 ± 0.0**
S35	1.7 ± 0.0**	0.7 ± 0.3**	0.5 ± 0.1	1.0 ± 0.4**	4.4 ± 0.3**
S29	1.0 ± 0.0	0.6 ± 0.0**	0.5 ± 0.0**	0.4 ± 0.0**	2.5 ± 0.0**
S42	4.2 ± 0.0**	<0.05	0.6 ± 0.0**	<0.05	4.8 ± 0.0**
S34	2.2 ± 0.0**	2.1 ± 0.1**	0.2 ± 0.0	3.0 ± 0.2**	7.5 ± 0.3**
S10	8.2 ± 0.4**	4.0 ± 0.0**	0.1 ± 0.0	2.2 ± 0.0**	14.6 ± 0.3**
S23	9.3 ± 0.7**	1.8 ± 0.3**	2.4 ± 0.1**	11.4 ± 1.0**	24.8 ± 0.6**
S7	26.5 ± 0.3**	2.6 ± 0.1**	1.4 ± 0.2**	13.9 ± 0.3**	44.4 ± 0.3**
S9	27.5 ± 0.6**	10.9 ± 0.7**	1.0 ± 0.1**	77 ± 0.7**	116 ± 1.2**
**CWt**	**0.7 ± 0.1**	**0.1 ± 0.0**	**0.3 ± 0.0**	**0.1 ± 0.0**	**1.2 ± 0.1**
C25	1.2 ± 0.0*	Traces	0.6 ± 0.0**	0.1 ± 0.0	2 ± 0.0**
C32	0.5 ± 0.1	0.1 ± 0.0	0.6 ± 0.1	0.1 ± 0.0**	1.4 ± 0.1
C26	0.4 ± 0.0	<0.05	0.6 ± 0.0**	<0.05	1 ± 0.0
C28	2.6 ± 0.0**	<0.05	0.1 ± 0.0	<0.05	2.7 ± 0.0**
C41	2.9 ± 0.2**	<0.05	0.2 ± 0.0	<0.05	3.2 ± 0.1**
C13	0.7 ± 0.0	33.5 ± 2.0**	2.5 ± 0.2**	0.2 ± 0.0*	36.8 ± 2.2**
C18	70.5 ± 2.9**	60.1 ± 1.8**	4.6 ± 0.3**	42.8 ± 1.7**	178 ± 6.2**
**DWt**	**0.2 ± 0.0**	**<0.05**	**0.4 ± 0.0**	**<0.05**	**0.6 ± 0.0**
D3	12.9 ± 0.2**	3.6 ± 0.0**	0.1 ± 0.0	3.5 ± 0.0**	20.2 ± 0.2**

Values are means of three independent seed samples; ± indicates SE. Comparisons were made per variety. *P ≤ 0.05, **P ≤ 0.01.

NPt, neopterin; MPt, monapterin; NPt‐G, neopterin glycoside; HMPt, 6‐hydroxymethylpterin.

**Table 2:** Breakdown pteridines in Pinto seeds

Pteridine content (nmol/g dry weight)				
	PtAl	Pt	CPt	Total breakdown
**SWt**	**ND**	**2.1 ± 0.1**	<0.05	**2.2 ± 0.1**
S30	1.9 ± 0.1	0.1 ± 0.0	<0.05	2.0 ± 0.1
S8	0.4 ± 0.0	1.7 ± 0.2	<0.05	2.1 ± 0.1
S35	0.2 ± 0.0	0.2 ± 0.1	<0.05	0.3 ± 0.1
S29	2.8 ± 0.1	0.2 ± 0.0	0.1 ± 0.0	3.0 ± 0.0**
S42	2.1 ± 0.1	0.1 ± 0.0	<0.05	2.2 ± 0.1
S34	ND	<0.05	0.1 ± 0.0	0.1 ± 0.0
S10	2.5 ± 0.0	<0.05	<0.05	2.5 ± 0.0*
S23	0.8 ± 0.0	15.7 ± 1.2**	<0.05	16.6 ± 1.3**
S7	6.6 ± 0.3	15.8 ± 0.8**	<0.05	22.4 ± 0.8**
S9	9.5 ± 1.0	0.1 ± 0.0	2.7 ± 0.3**	12.3 ± 0.9**
**CWt**	**ND**	**3.1 ± 0.2**	**0.1 ± 0.0**	**3.2 ± 0.2**
C25	0.1 ± 0.0	0.7 ± 0.0	<0.05	0.8 ± 0.0
C32	2.5 ± 0.2	<0.05	<0.05	2.5 ± 0.1
C26	2.1 ± 0.2	0.8 ± 0.0	<0.05	3.0 ± 0.1
C28	0.9 ± 0.0	1.0 ± 0.0	<0.05	1.9 ± 0.1
C41	1.0 ± 0.0	0.6 ± 0.1	<0.05	1.6 ± 0.1
C13	1.1 ± 0.1	3.3 ± 0.2	<0.05	4.4 ± 0.1**
C18	57.8 ± 2.8	3.5 ± 0.3	14 ± 1.4**	75.1 ± 3.5**
**DWt**	**ND**	**0.8 ± 0.0**	**<0.05**	**0.8 ± 0.0**
D3	2.2 ± 0.0	<0.05	<0.05	2.3 ± 0.0**

Values are means of three independent seed samples; ± indicates SE. Comparisons were made per variety. *P ≤ 0.05, **P ≤ 0.01.

PtAl, pterin aldehyde; Pt, pterin; CPt, 6‐carboxypterin.

### AtGCHI expression significantly augments biosynthetic pteridines in T_3_ bean seeds

**Figure 4:**
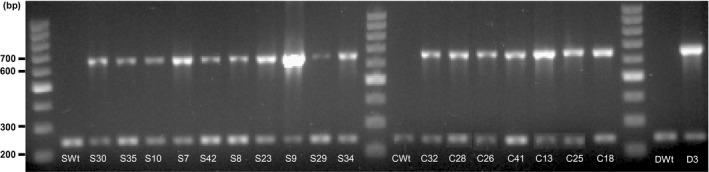
*AtGchI* expression in Pinto bean T_3_ seeds. RT‐PCR of total RNA using specific primers for *AtGchI* (607 bp) using *Pv18s* as control (225 bp). S (Saltillo), C (Café) and D (Durango).

The expression of recombinant *AtGchI* was evaluated by RT‐PCR. Transgene expression was detected in all the transformed seeds, while Wt controls did not show any amplification (Figure 4). Pteridine characterization in AtGCHI^+^T_3_ seeds showed marked increases in all pteridine forms observed in Wt seeds, 6‐carboxypterin (Cpt), NPt, Mpt, Pt and HMPt (Figure 3c, Table 1). Moreover, pterin 6‐aldehyde (PtAl), a product of folate breakdown (Orsomando *et al*., 2006), was detected in transgenic lines but it was below our detection limits in Wt seeds (Table 2). PtAl appeared in all but one transgenic line, even in those that did not demonstrate significant increases in total pteridine contents. Interestingly, in the majority of the low‐pteridine lines, the pteridine profile was shifted away from a mainly Pt accumulation in Wt towards PtAl in AtGCHI seeds (Table 2). Pt was significantly increased in only a few lines, and Cpt, the most oxidized pteridine product (Figure 1b), was present in trace amounts and only showed significant increases in the pteridine hyperaccumulators (S9 and C18). When considering biosynthetic pteridines, low‐pteridine accumulators had increases in Npt and only three lines accumulated significant amounts of Mpt (S35, S29 and S34). From this group, S35 and S34 also showed higher HMPt than Wt controls. Transgenic lines ordered according to their increasing amount of total pteridines show that all biosynthetic pteridines were enhanced considerably (Table 1). In general, Npt was the most abundant species, whereas breakdown pteridines did not accumulate proportionally (Table 2). These results show that AtGCHI overexpression caused significantly higher levels of total pteridines; moreover, the biosynthetic/breakdown ratio was shifted towards a predominant contribution of biosynthetic species (6.9 on average), while in the Wt seeds this ratio was 0.57.

### AtGCHI overexpression increases folate contents in T_3_ seeds

**Figure 5:**
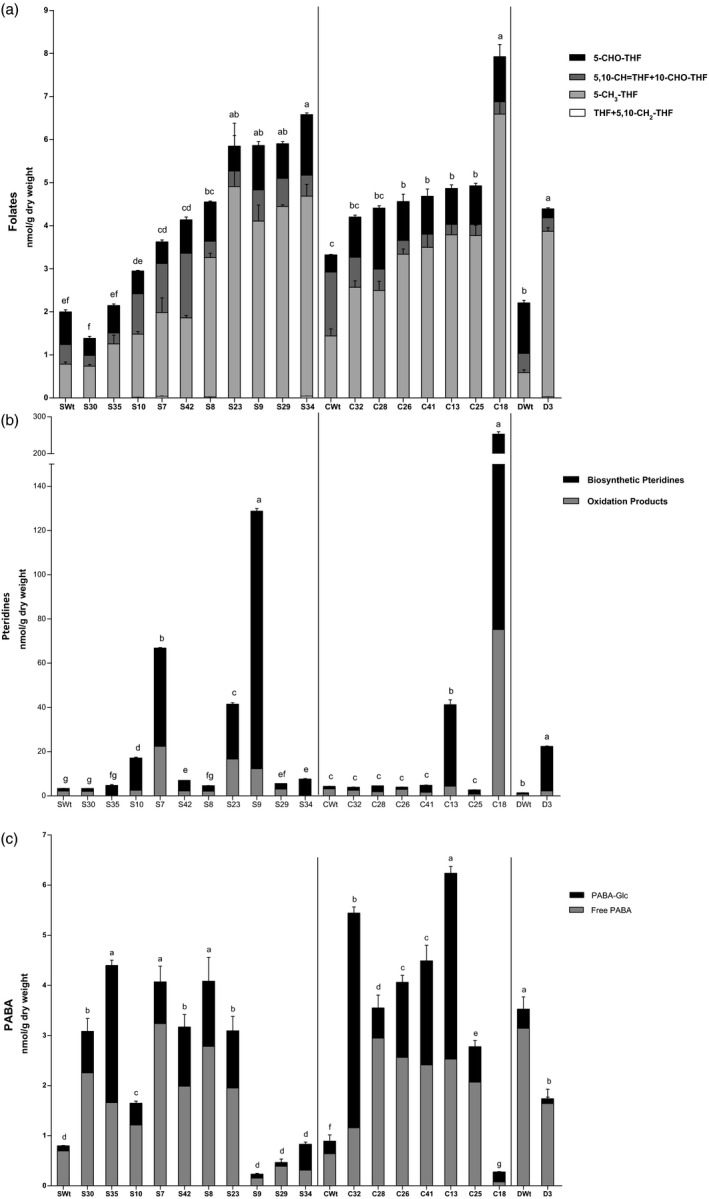
Accumulation of folates and precursors in AtGCHI‐expressing T_3_ seeds. Folate species (Panel a), pteridines (Panel b) and PABA (Panel c) contents. Controls are Wt seeds from each variety. Values are means of three independent seed samples; error bars indicate SE. Different letters indicate statistical difference using Student's *t*‐test (*P* < 0.05). Comparisons were made per variety.

Folate levels increased in the three transformed cultivars as a result of pteridine overproduction (Figure 5a). Pinto Saltillo‐independent overexpressors displayed increases in total folate contents ranging from 1.5 to 3.3 times the SWt levels with up to 6.6 nmol/g DW. Pinto Café AtGCHI seeds showed modest but significant increases in total folates, ranging from 37% to 48% higher than the CWt. However, C18, the line that hyperaccumulated pteridines, also had the highest folate content with 7.9 nmol/g DW. This represented a 2.4‐fold increase when compared to CWt. In addition, the Pinto Durango D3 line doubled the amount of this vitamin when compared to the control. It is worth noting that the lines that accumulated more pteridines were not always the lines that had the highest folate levels (Figure 5a and b).

With regard to the folate classes, the majority of the increases in total folates were due to increases in the predominant folate form in bean seeds –5‐CH_3_‐THF– which accumulated up to 6.5 times the levels that were found in their Wt counterparts. In contrast, THF was barely detectable in some lines and it was not detected in all Café samples. 5‐CHO‐THF increased modestly by up to 3.5‐fold and 5,10‐CH=THF (which, in our analyses, includes 10‐CHO‐THF) decreased in some of the lines when compared to the Wt folate profiles (Figure 5a). A more evident change in the distribution of folate classes was observed in the Pinto Durango overexpressor. In this case, the increase in folate levels caused a change in the profile, favouring 5‐CH_3_‐THF accumulation with a 553% increase while the other two species sharply decreased.

Folates usually have a polyglutamyl chain that influences coenzyme affinity and intracellular transport (Hanson and Gregory, 2011). The degree of polyglutamylation of 5‐CH_3_‐THF in bean seeds in Wt lines and AtGCHI^+^T_3_ showed very similar profiles regardless of the variety. Supplementary Figure S2 depicts the polyglutamylation profiles of the lines that accumulated more folates. The predominant folate form was pentaglutamylated, representing >66% of the 5‐CH_3_‐THF pool in all the bean seeds analysed; mono‐ and hexaglutamyl forms comprised the rest of the pool. Although 5‐CH_3_‐THF decaglutamate was barely detected in the Café and Durango Wt varieties, this folate derivative increased significantly in biofortified seeds: 22‐ and 84‐fold, respectively (C18 and D3).

### AtGCHI overexpression causes an up‐regulation of PABA synthesis

PABA, the other folate precursor, occurs in plant cells as free and esterified to glucose (PABA‐Glc); free PABA is able to cross membranes to participate in folate synthesis, while PABA‐Glc needs to be hydrolysed first and it is believed to be a form of storage (Quinlivan *et al*., 2003). Total PABA levels differed among the Wt bean varieties. Pinto Durango seeds accumulated the highest amount, 3.5 nmol/g DW, while Saltillo and Café seeds had five times less. Surprisingly, many of the Saltillo and Café AtGCHI overexpressors accumulated significantly more PABA than Wt seeds, even those that did not show significant pteridine increases (Figure 5c). We only observed similar or less PABA than Wt in some lines that had the highest folate levels. However, if we take into account the PABA moiety contained in the folate molecule (total sum of PABA), all Saltillo and Café lines accumulated higher PABA than Wt seeds, and only D3 had equal levels of the total sum of PABA as DWt (Supplementary Figure S3). Regarding PABA distribution, the majority of the pool was in its free form in all Wt varieties (>72%, Figure 5c). By contrast, the proportion of PABA‐Glc was higher than free PABA in the AtGCHI lines; this is not surprising whether we consider it to represent the storage form of the folate precursor.

The increases in the total sum of PABA after pteridine engineering were unexpected, as GCHI overexpression had never caused an increase in PABA levels in the other engineered plants. This increase was also witnessed in T_4_ bean seeds (Supplementary Figure S4a). Thus, to evaluate whether PABA synthesis was enhanced as a result of AtGCHI overexpression at the transcriptional level, we analysed the expression of the endogenous gene *PvAdcs* in selected lines. This gene codes for the enzyme that catalyses the first step for PABA synthesis (Figure 1b, enzyme C). *PvAdcs* expression was detected in all of the analysed samples, in both the Wt and AtGCHI lines (Supplementary Figure S4b). *PvAdcs* expression increased slightly in three of the analysed AtGCHI lines while the other five expressed the same level as the Wt seeds. S8 and S34 seeds had 30% and 34% higher *PvAdcs* expression than SWt, and C25 expressed this endogenous gene 2.5 times more than CWt. These results show that an up‐regulation of *PvAdcs* could have happened as a consequence of the engineering, but as we did not observe a clear correlation between *PvAdcs* expression and PABA increase, this result was probably caused by additional and unknown modifications in its production.

**Figure 6:**
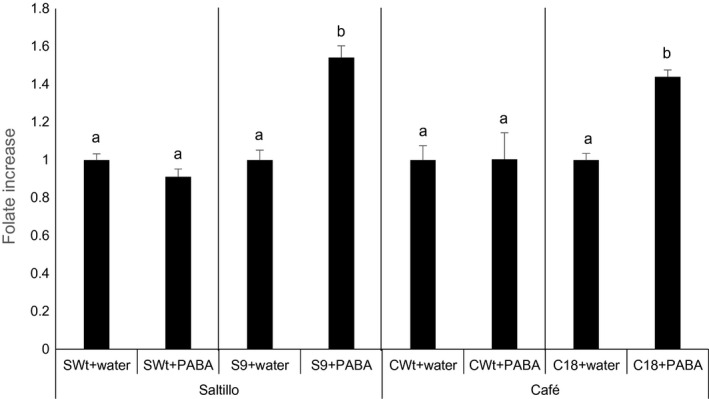
Folate increases in selected AtGCHI bean seeds imbibed with PABA. Pinto bean seeds were imbibed in water as controls or PABA, folate fold‐changes are shown in relation to Wt+ water controls. Values are means of three independent seed samples; error bars indicate SE. Letters indicate statistical difference using Student′s *t*‐test (*P* < 0.05). Comparisons were made per variety.

Overall, the analysis of folates and their precursors not only shows an up‐regulation of PABA production but also indicates the existence of a bottleneck downstream in the folate biosynthetic pathway: both pteridines and PABA still accumulated in some lines (e.g. S7, S23, C13). Thus, we questioned whether PABA engineering could enhance folate production as it had in other plants. To determine this, PABA was fed to the seeds (Wt and selected AtGCHI lines) by imbibition. A 0.2 mm PABA solution was fed for 12 h to the S9 and C18 lines, which were high in both folates and pteridine contents but had low free PABA levels. As folates increase in legume seeds when the seed dormancy is released by imbibition (Sahr *et al*., 2005), controls included seeds imbibed in water only. Folates were quantified, and we compared PABA‐fed seeds against those imbibed in water (Figure 6). For the two Wt varieties analysed, there was no change in the folate contents of the seeds imbibed in water and those imbibed a PABA solution; by contrast, in both AtGCHI overexpressors S9 and C18, the folate contents increased by 54% and 44%, respectively. Even though the feeding was carried out by the imbibition of the already desiccated seed, these results suggest that increasing PABA production in the developing AtGCHI seeds could further increase their folate levels.

## Discussion

Folates can be engineered in a good folate source, common bean seeds, by overexpressing a committed step of the biosynthetic route. This work shows the engineering of folates in bean seeds from three different Mexican cultivars. To our knowledge, this is the first report of a *P. vulgaris* plant from a Mexican cultivar being stably transformed and the first biofortification with folates in a leguminous species. In this work, we overexpressed the pteridine branch of the folate biosynthesis pathway as it had been done in other plants (Diaz de la Garza *et al*., 2004; Hossain *et al*., 2004; Naqvi *et al*., 2009; Nunes *et al*., 2009; Storozhenko *et al*., 2007). In accordance with previous studies, this strategy led to modest increases in folate levels. Common bean already contains significant amounts of folate; however, the 3.3‐ and 2.4‐fold increases in the Saltillo and Café lines resulted in values as high as 268 and 325 μg of folate in a 100 g portion, which represents 67% and 82%, respectively, of the recommended daily allowance (RDA) for an adult. It is noteworthy that with exception of rice, in which folate levels were not changed, augmenting only the pteridine content in fruit, leaves, tubers and endosperm yielded very similar increases in each case (between twofold and fourfold). This occurred regardless of the initial folate levels of the Wt species, which in each case varied from very low in cereals (0.5 nmol/g) to high in leaves and bean seeds (close to 3 nmol/g). Additionally in the aforementioned studies, the polyglutamylation degree of 5‐CH_3_‐THF did not change after folates were only doubled, contrary to what was observed when folates were boosted to higher levels and monoglutamylated forms were predominant (Diaz de la Garza *et al*., 2007; Storozhenko *et al*., 2007). In the present engineering work, the distribution of the polyglutamylated forms of 5‐CH_3_‐THF in some AtGCHI lines slightly differed from the Wt in their proportion of 5‐CH_3_‐THF decaglutamate (Supplementary Figure S2). This could be explained by differences in FPGS activities during bean seed development when compared to those from other tissues and plant species.

### Analyses of folate precursors in common bean seeds suggest distinct regulation of folate homoeostasis in this legume seed

Pteridines found in Wt common bean seeds accumulated in higher amounts than those measured previously in other engineered plants. For example, Saltillo and Café seeds had 7 and 11 nmol/g of DW (5.7 and 9 nmol/g of FW basis), while tomato fruit, rice endosperm and potato tubers had very small amounts <1 nmol/g of FW. High pteridine accumulation seems common in legume seeds; very early research in this area quantified pteridines in seeds from kidney (*P. vulgaris*) and Adzuki beans (*Vigna angularis*) which had 6.3 and 17.3 nmol/g DW, respectively (Kohashi *et al*., 1980). However, the majority of the accumulated pteridines characterized here were not folate precursors (Tables 1 and 2); therefore, even when Wt seeds had high pteridine levels, the majority of the pteridine pool was probably the result of folate degradation.

Folate breakdown in plants occurs at calculated rates of 10% per day, which is considered higher than in mammalian systems (Hanson and Gregory, 2011). Folate breakdown products include PtAl, CPt (Orsomando *et al*., 2006), and probably Pt, which could come from Cpt decarboxylation, which occurs under UV‐light radiation *in vitro* (Lowry *et al*., 1949). Thus, the high accumulation of breakdown pteridines in Wt bean seeds suggests a high rate of folate breakdown in desiccating seeds. The pteridine profile in engineered seeds supports this as a boost in pteridine production caused modest increases in folate and breakdown pteridines. However, it could be argued that for the engineered seeds, all these pteridines could be the product of pteridine oxidation. HMPt can be photo‐oxidized to PtAl in solution (Rokos and Schallreuter, 2002), and sepiapterin is oxidized to CPt when human epidermal cells are irradiated (Rokos *et al*., 2002); however, information regarding pteridine degradation is lacking for plants. Even when this is a possibility, we consider it unlikely, based on the following considerations. Plant cells could protect pteridines from photo‐oxidation with light harvesting, antioxidant, or reducing compounds or even through the enzymes that utilize them for folate biosynthesis. If the observed oxidized pteridines were the product of pteridine photo‐oxidation, then the highest pteridine overproducers would have greater proportions of oxidized pteridines. However, this was not the case. S9 and C18, the highest pteridine producers, had just 10% and 30% breakdown pteridines, which contrasts to the Wt proportions (65% on average). Tomato fruit that hyperaccumulated pteridines and presented modest folate increases showed a similar behaviour: HMPt was prevalent and no PtAl or Pt were detected (Diaz de la Garza *et al*., 2004).

On the other hand, if these pteridines were in their majority folate breakdown products, this would imply that the modest increase in folate levels observed in some lines would lead to a concomitant increase in folate breakdown. The presence of PtAl in almost all engineered lines suggests that this might be happening. It is important to clarify that we did not assess the reduction level of pteridines as they need to be fully oxidized to be detected. Therefore, the PtAl that we observed could be in part reduced in the cell as dihydropterin aldehyde (Figure 1b, DHPtAl). DHPtAl is reduced to HMDHPt, by an aldehyde reductase (Figure 1b, enzyme J) as a folate salvage reaction. In fact, this enzyme has been previously characterized and its activity in seeds was found to be significantly lower than in leaves (Noiriel *et al*., 2007). Thus, it is tempting to speculate that the rate of folate turnover in desiccating bean seeds might be high and assume that the folate salvage system is not coping with the increased flux due to the engineering. Therefore, regulation of folate homoeostasis during seed desiccation and postharvest handling becomes critical for engineering folates in legume seeds. The results from the folate engineering in rice endosperm support this assumption, as engineered rice grains had high rates of folate losses during seed storage (Blancquaert *et al*., 2015).

It should be noted that the biosynthetic pteridines measured in AtGCHI seeds did not always correlate with the amount of folates. This lack of correlation is not surprising, as it was already observed when pteridines were enhanced in Arabidopsis leaves, tomato fruit, rice endosperm and potato tubers (Blancquaert *et al*., 2013; Diaz de la Garza *et al*., 2004; Hossain *et al*., 2004; Storozhenko *et al*., 2007). There are two plausible explanations for this; first, if the measured pteridines were not in the dihydro form, they could not be used for folate biosynthesis. Second, if HMDHPt is not within the mitochondria, its phosphorylation and conjugation with PABA to form dihydropteroate cannot occur (DHP, Figure 1b). These would hamper a direct correlation between measured pteridines and folates. In fact, other engineering study has considered pteridine transport into mitochondria as a possible downstream bottleneck for folate biosynthesis (Diaz de la Garza *et al*., 2007).

### PABA production is induced by AtGCHI expression in common bean seeds

Unexpectedly, the total sum of PABA was significantly increased in all the transgenic seeds but D3 when compared to their Wt counterparts (Supplementary Figure S3). We explored the possibility that AtGCHI expression triggered PABA synthesis at the transcriptional level, in particular towards the expression of the endogenous *PvAdcs* that codes for the first step for PABA biosynthesis in plastids (Figure 1b, enzyme C). The results, while inconclusive, do not rule out a possible regulation through the expression of this gene. We measured the *PvAdcs* expression in the already desiccated seed; therefore, we cannot know its dynamics throughout the seed's development when AtGCHI begins to be expressed by means of the β‐conglycinin promoter (Chen *et al*., 1989). More thorough experiments need to be conducted to address this question. Nevertheless, we were able to provide evidence to show that the response of the cell in the case of common bean seeds was different than that observed in other engineered plants. PABA levels almost disappeared in tomato fruit when pteridines were increased due to its use for folate biosynthesis (Diaz de la Garza *et al*., 2004) and ADCS expression was not affected (Waller *et al*., 2010). For rice endosperm, PABA pools also decreased in pteridine overproducers (Storozhenko *et al*., 2007) while folate biosynthesis was not altered transcriptionally after rice biofortification (Blancquaert *et al*., 2013). In potato tubers, endogenous *GchI* and *Adcs* expression had high variations in AtGCHI overexpressors hinting at an endogenous response; however, PABA decreased in immature AtGCHI potato tubers (Blancquaert *et al*., 2013). All these works highlight the differences in folate regulation among plant tissues. Here, we show that PABA production was enhanced in engineered AtGCHI seeds, even in those that show subtle pteridine increases. The question that still remains is which mechanism(s) and molecule(s) could be responsible for this elicitation.

These results also show that there are additional limiting steps downstream of the two committed folate biosynthetic steps for common bean seeds. In some transgenic lines, PABA and biosynthetic pteridines accumulated enough to synthesize more folates. As previously mentioned, as folate biosynthesis is highly compartmentalized it is not possible to point out an exact bottleneck without knowing the subcellular pools. However, when PABA was fed to seeds that hyperaccumulated pteridines, we could further increase folates (Figure 6). The simultaneous enhancement of PABA and pteridines during seed development will show the limits of the two‐gene strategy for bean seeds; in addition, it seems that a pull strategy might also result in more folate accumulation. This approach was recently successfully used to stabilize boosted folates by overexpressing the cytosolic FPGS plus a folate‐binding protein for folate ‘sequestration’ in rice endosperm (Blancquaert *et al*., 2015).

### Folates engineered in bean seeds could contribute to alleviate folate malnutrition

The folate that was predominantly enhanced was 5‐CH_3_‐THF, even when the initial folate composition was slightly different among the Wt seeds. 5‐CH_3_‐THF is the circulating folate in the bloodstream, and its bioavailability has been proven in humans. Also, it has been suggested that it is a better folate source than folic acid, as it is already fully reduced (Lamers *et al*., 2006; Pietrzik *et al*., 2010). Thus, folates engineered in common bean grains have the potential to increase folate status in humans, if consumed, as demonstrated in biofortified tomato fruit and rice endosperm (Castorena‐Torres *et al*., 2014; Kiekens *et al*., 2015). On the other hand, pteridine build‐up has been a concern for human consumption, as HMPt is not part of the human pteridine metabolism. However, the pteridines characterized in Wt beans show that we have been exposed to relatively high pteridine levels through common bean consumption, especially in Latin American countries, in which this legume is a staple crop.

## Conclusions

Regulation of folate metabolism in plants has been difficult to elucidate because it is highly compartmentalized, folate and pteridine chemistries and liabilities make their accurate quantification challenging, and also because of the significant differences in folate homoeostasis among plant tissues. Metabolic engineering in different food crops has aimed at increases in folate pools and, in addition, is currently providing important insights into folate regulation. This particular study shows that folate metabolism might be differentially regulated in this legume in comparison with other crops and plant organs. We also show that an already good source of folate can be engineered to accumulate even higher levels of this vitamin. This is relevant because approximately half of the folates in legumes suffer from degradation and leaching as a result of the cooking process (Dang *et al*., 2000). Further work on folate engineering and its dynamics during postharvest handling will make an important contribution to the current limited knowledge on folate homoeostasis in plants. This knowledge is necessary to design strategies to improve folate levels through engineering or by breeding and maintaining their levels throughout the food supply chain. Common bean is the most consumed legume in the world; it is cultivated as a subsistence crop by rural populations in which folate fortification efforts are difficult to implement. Moreover, it is a good source of several vitamins and it is already the subject of important biofortification efforts with iron and zinc (Mayer *et al*., 2008), making it an excellent crop for delivering nutrients. In this work, we confirmed that common bean plants can be stably transformed and that we can use engineering strategies to increase folate levels in the edible seeds. However, further engineering is needed to achieve folate levels that are capable of providing an adult's entire RDA in a single serving of cooked common beans. This would help to lower the current number of 300 000 annual cases of NTD (Berry *et al*., 2010) and impact the other thousands of cases of people with health problems related to folate deficiency worldwide.

## Experimental procedures

### Plant material

Pinto Saltillo (1424‐FRI‐026‐120901/C), Pinto Café (wild variety) and Pinto Durango (PT91082) cultivars were donated by Dr. Jorge Acosta Gallegos (INIFAP) and were grown under greenhouse conditions and subjected to standard fertilization and pest control measurements. Seeds were harvested from dried pods and kept at 4 °C until transformation. T_2_, T_3_ and T_4_ seeds were grown and harvested under the same conditions and stored at −20 °C for further characterization.

### Construction of the pAHAS‐AtGCHI vector

A 2.5‐kb AtGCHI expression cassette containing the *AtGchI* gene (AT3G07270), β‐conglycinin promoter (M13759.1) and *35S* terminator was constructed by PCR‐SOEing using the pVT103u vector as a template for the *AtGchI* gene (Basset *et al*., 2002), and a pβ‐Cong vector was used as a template for the promoter and terminator sequences (Vianna *et al*., 2011). The plant consensus leader sequence TAAACA was added before the start codon (Koziel *et al*., 1996). The AtGCHI expression cassette was subcloned into a pGEM T‐Easy vector (Promega, Madison, WI) and sequenced. The pAHAS plasmid was linearized with NotI and ligated with the AtGCHI cassette to produce pAHAS‐AtGCHI, which was used for the transformation. The pAHAS contains the *Arabidopsis thaliana* acetohydroxy acid synthase gene (x51514) for resistance to imidazoline herbicides (Sathasivan *et al*., 1990).

### Common bean transformation

Common bean seeds were imbibed for 12 h in water to soften the tissues and embryos were excised from cotyledons. Apical meristems were exposed by cutting out the primordial leaves, and then transformed by particle bombardment. The protocol used is described elsewhere (Rech *et al*., 2008) with minor modifications. Briefly, tungsten particles were coated with 8 μg of pAHAS‐AtGCHI linearized with KpnI and used for embryo bombardment utilizing a high‐pressure Helium microparticle acceleration system (IPS Ltd, Brazil). After biolistic transformation, embryonic axes were placed on the elongation medium 1 (Murashige and Skoog medium containing 44.3 μm 6‐Benzylaminopurine [BAP], 80 nm Imazapyr, as a selection marker, 0.6% agar, pH 5.7) for 1 week. Then, embryonic axes were transferred to the elongation medium 2 (medium 1 without BAP) until they reached a convenient size for acclimation.

### Screening and acclimation of transgenic plants

Imazapyr‐resistant plants were screened for the *AtGchI* and *ahas* sequences by PCR. Genomic DNA was extracted from leaves with a CTAB DNA buffer (2% CTAB, 1.4 m NaCl, 100 mm Tris–HCl pH 8.00, 20 mm EDTA and 1% PVP) and used as a template for PCR (Supplementary Table S1). Plants positive for the *AtGchI* and *ahas* genes that grew around 10 cm high were transplanted and acclimated into a mixture of soil and vermiculite (1:1). PCR was performed in more leaves as the plants developed, and seeds from positive plants also were screened.

### Folate analysis

Folates were extracted and quantified as previously described (Ramos‐Parra *et al*., 2013) with some modifications. Seeds (0.5 g) were ground in liquid nitrogen, and the extract was treated for 2 h with protease (Sigma, St. Louis, MO). Half of the extract was used for the determination of monoglutamylated folates and the other half for polyglutamates. Folates were purified by affinity chromatography and analysed using an HPLC‐electrochemical detector (CoulArray Model 5600A; ESA, Chelmsford, MA). Separation was achieved with an Atlantis dC18 (150 × 4.6 mm, 5 μm Waters, Milford, MA) column using a nonlinear gradient of mobile phase A (28 mm K_2_HPO_4_, 59 mm H_3_PO_4_) and B (75% phase A, 25% acetonitrile); detection was achieved with an electrochemical cell adjusted to 100, 200, 300 and 400 mV, and quantification was made using THF, 5‐methyl‐THF, 5, 10‐methenyl‐THF and 5‐formyl‐THF standards (Schircks, Jona, Switzerland).

### Pteridine analysis

Pteridine contents were quantified as previously described (Diaz de la Garza *et al*., 2004). Seeds (0.3 g) were ground in liquid nitrogen; pteridines were extracted using a chloroform/methanol/water mixture and cleaned by phase partition; and the extracts were oxidized by I_2_/KI in HCl and excess of I_2_ was removed with ascorbate. Extracts were separated by HPLC with a 4‐μm, 250 × 4.6‐mm Synergy Fusion‐RP 80 column (Phenomenex, Torrance, CA), elution was isocratic with a mobile phase of 10 mm Na‐phosphate (pH 6.0) at 1.0 mL/min. Detection was achieved by a Waters 2475 fluorescence detector (350‐nm excitation and 450‐nm emission), and chromatographic peaks were identified by comparing them to standards obtained from Schircks (Jona, Switzerland). To assess conjugated pterins, 2 m HCl was added to one‐half of the extract and incubated at 80 °C for 1 h for full hydrolysis.

### PABA analysis

PABA contents were determined as previously described (Quinlivan *et al*., 2003) with some modifications. 0.3 g of seeds was extracted twice in methanol (2 mL). Extracts were divided into two and dried: one‐half was used for the determination of free PABA (resuspended in water), and the other half was used to assess conjugated PABA resuspension which was done in 0.1 m HCl for acid hydrolysis. Extracts were cleaned by ion exchange followed by ethyl acetate partition as described. Forty μL of PABA extract was injected onto a 5‐μm, 250 × 3 mm Luna C_18_ column (Phenomenex) and eluted isocratically with 0.5% acetic acid: methanol (80:20, v/v) at a flow rate of 1 mL/min. PABA was detected using a Waters 2475 fluorescence detector (290‐nm excitation, 340‐nm emission) and quantified the PABA standard (Sigma). Conjugated PABA was determined by subtracting the nonhydrolysed PABA value from the total PABA.

### PABA imbibition

Seeds from SWt, CWt, S9 and C18 (5 g each) were imbibed for 12 h in 10 mL of 0.2 mm PABA standard solution or Milli‐Q water as a control, following incubation at room temperature for 12 h on a paper towel. The moisture content (AOAC, 1995) was determined for each treatment after incubation, and the rest of the samples were stored at −20 °C until folate analysis.

### Total RNA extraction, cDNA synthesis, RT‐PCR and RT‐qPCR

For RT‐PCR, total RNA was extracted from seeds with CTAB RNA buffer (2% CTAB, 2 m NaCl, 100 mm Tris–HCl pH 8.0, 25 mm EDTA, 0.1% spermidine, 2% β‐mercaptoethanol and 2% PVP) as described (Sangha *et al*., 2010). RNA was treated with Turbo DNA‐Free Kit (Invitrogen, Carlsband, CA) to remove genomic DNA. Then, cDNA was synthetized using SuperScript III (Invitrogen). For RT‐qPCR, total RNA was isolated from the seeds (200 mg) in CTAB buffer and RNA was cleaned as described (Morante‐Carriel *et al*., 2014). RT‐qPCR was performed on a Rotor Gene RG‐3000 thermal cycler (Corbett Research, Sydney, Australia) using the Brilliant III Ultra‐Fast SYBR Green QPCR Master Mix (Agilent Technologies, Santa Clara, CA). Relative expression analysis was performed according to the 2^∆∆Ct^ method (Livak and Schmittgen, 2001). PCR primers and conditions are shown in Supplementary Table S2.

### Statistical analyses

All determinations were conducted in triplicate, and the results were expressed as the mean ± standard error (SE). Statistical analyses were conducted by one‐way ANOVA, and mean differences were calculated using Student's *t*‐test. A level of significance of 0.05 was used in all tests, using JMP^®^ Version 5 software (SAS Institute Inc., Cary, NC).

## Supporting information


**Figure S1.** Diagram of the vector used for the transformation of common bean.
**Figure S2.** Polyglutamyl profile of 5‐CH_3_‐THF in selected Pinto bean seeds.
**Figure S3.** Total sum of PABA in AtGCHI expressing T_3_ seeds.
**Figure S4.** Accumulation of PABA and expression of *P. vulgaris aminodeoxichorismate synthase (PvAdcs)* in selected AtGCHI expressing T_4_ seeds.
**Table S1.** Primers used to screen the expression cassettes in transformed plants.
**Table S2.** Oligonucleotide sequences used for RT‐PCR and RT‐qPCR analysis.
