# Supplementary material for: Metabolic engineering of folate and its precursors in Mexican common bean (Phaseolus vulgaris L.)
Source: Plant Biotechnol J. 2016 Apr 25;14(10):2021–32. doi: 10.1111/pbi.12561 (PMC5043471; doi:10.1111/pbi.12561)
Supplement: Supplementary file 1 — Figure S1. Diagram of the vector used for the transformation of common bean. Figure S2. Polyglutamyl profile of 5‐CH3‐THF in selected Pinto bean seeds. Figure S3. Total sum of PABA in AtGCHI expressing T3 seeds. Figure S4. Accumulation of PABA and expression of P. vulgaris aminodeoxichorismate synthase (PvAdcs) in selected AtGCHI expressing T4 seeds. Table S1. Primers used to screen the expression cassettes in transformed plants. Table S2. Oligonucleotide sequences used for RT‐PCR and RT‐qPCR analysis. [file PBI-14-2021-s001.docx]

**Supplementary Table 1.** Primers used to screen the expression cassettes in transformed plants. Forward and reverse primers anneal to the promoter and to the transgene respectively.

| Gene | Accession Number | Sequence (5'-3') | Direction | Annealing  Temperature (°C) | Size (bp) |
| --- | --- | --- | --- | --- | --- |
| *Gm*β-*conglycinin* | M13759.1 | CGCAATCACACACAGTGG | Forward | 56 | 1028 |
| *AtGchI* | AT3G07270 | CCGAGGAAACCAGTAGCT | Reverse |  |  |
| *AtAhas promoter* | AL133315.1 | TCGTAATCGAACGCGTTAC | Forward | 56 | 970 |
| *AtAhas* | X51514.1 | GCAGCTTGTATCCATTCTC | Reverse |  |  |

PCR conditions: One cycle at 5 min at 95° C, 35 amplification cycles (95°C for 1 min, 56°C for 1 min, 72°C for 1 min), final cycle of 5 min at 72°C

**Supplementary Table 2.** Oligonucleotide sequences used for RT-PCR and RT-qPCR analysis.

| Gene | Accession Number | Sequence (5'-3') | Direction | Annealing Temperature (°C) | Size (bp) |
| --- | --- | --- | --- | --- | --- |
| *AtGchI* | AT3G07270 | GCGGCCGCCTCGAGGTTTTCAAATTTGAAT | Forward | 56 | 607 |
|  |  | CCGAGGAAACCAGTAGCT | Reverse |  |  |
| *PvAdcs* | KM668035 | CTTTCTCTGCTCATGCCATT | Forward | 56 | 100 |
|  |  | TTCCTCTAGTGATCAGTGGC | Reverse |  |  |
| *Pv18s* | YP_001122828 | TCGAAGCGATCTTTTCGTAG | Forward | 56 | 300 |
| *(housekeeping)* |  | TTCTCAGTCGACTCGCTTTTT | Reverse |  |  |

RT-PCR conditions: One cycle at 5 min at 95° C, 35 amplification cycles (95°C for 1 min, 56°C for 1 min, 72°C for 1 min), final cycle of 5 min at 72°C. RT-qPCR conditions: One cycle at 10 min at 95° C, 40 amplification cycles (95°C for 10 s, 56°C for 15 s, 72°C for 22 s), final melting cycle from 72 to 95°C.

**Supplementary Figure 1.** Diagram of the vector used for the transformation of common bean. AtGCHI expression cassette is flanked by NotI sites, *AtGchI* expression is driven by the β-Conglycinin promoter.

**Supplementary Figure 2.** Polyglutamyl profile of 5-CH_3_-THF in selected Pinto bean seeds. Values are from the Wt and AtGCHI expressing lines with the highest content of total folates. Glu 9 was not detected in any extract. Values are means of three independent seed samples; error bars indicate SE.

**Supplementary Figure 3.** Total sum of PABA in AtGCHI expressing T_3_ seeds. Total sum of PABA includes free PABA, PABA-Glucosylated, and the PABA moiety within the folate molecule. Controls are Wt seeds from each variety. Values are means of three independent seed samples; error bars indicate SE. Different letters indicate statistical difference using Student’s *t*-test (*P<0.05*). Comparisons were made per variety.

**Supplementary Figure 4.** Accumulation of PABA and expression of *P. vulgaris aminodeoxichorismate synthase* (*PvAdcs*) in selected AtGCHI expressing T_4_ seeds. PABA content (Panel A) and RT-qPCR analysis (Panel B). Controls are Wt seeds from each variety. Values are means of two independent seed samples for PABA levels and for relative expression analysis n=3. Error bars indicate SE. Different letter indicates statistical difference using Student’s *t*-test (*P*<0.05). Comparisons were made per variety.
